# Population genetic structure and demographic history reconstruction of introduced flathead catfish (*Pylodictis olivaris*) in two US Mid‐Atlantic rivers

**DOI:** 10.1111/jfb.15888

**Published:** 2024-08-12

**Authors:** Justin M. Waraniak, Michael S. Eackles, Jason Keagy, Geoffrey D. Smith, Megan Schall, Sydney Stark, Shannon L. White, David C. Kazyak, Tyler Wagner

**Affiliations:** ^1^ Pennsylvania Cooperative Fish and Wildlife Research Unit, Department of Ecosystem Science and Management The Pennsylvania State University University Park Pennsylvania USA; ^2^ U.S. Geological Survey Eastern Ecological Science Center Kearneysville West Virginia USA; ^3^ Department of Ecosystem Science and Management The Pennsylvania State University University Park Pennsylvania USA; ^4^ Pennsylvania Fish and Boat Commission Bellefonte Pennsylvania USA; ^5^ Pennsylvania State University, Biological Services Hazleton Pennsylvania USA; ^6^ U.S. Geological Survey, Pennsylvania Cooperative Fish and Wildlife Research Unit Pennsylvania State University University Park Pennsylvania USA

**Keywords:** approximate Bayesian computation, genetic bottlenecks, invasive species, microsatellites, riverscape genetics, Susquehanna River

## Abstract

Population genetic analysis of invasive populations can provide valuable insights into the source of introductions, pathways for expansion, and their demographic histories. Flathead catfish (*Pylodictis olivaris*) are a prolific invasive species with high fecundity, long‐distance dispersal, and piscivorous feeding habits that can lead to declines in native fish populations. In this study, we analyse the genetics of invasive *P. olivaris* in the Mid‐Atlantic region to assess their connectivity and attempt to reconstruct the history of introduced populations. Based on an assessment across 13 microsatellite loci, *P. olivaris* from the Susquehanna River system (*N* = 537), Schuylkill River (*N* = 33), and Delaware River (*N* = 1) have low genetic diversity (global *H*_obs_ = 0.504), although we detected no evidence of substantial inbreeding (*F*_IS_ = −0.083 to 0.022). *P. olivaris* from these different river systems were genetically distinct, suggesting separate introductions. However, population structure was much weaker within each river system and exhibited a pattern of high connectivity, with some evidence of isolation by distance. *P. olivaris* from the Susquehanna and Schuylkill rivers showed evidence for recent genetic bottlenecks, and demographic models were consistent with historical records, which suggest that populations were established by recent founder events consisting of a small number of individuals. Our results show the risk posed by small introductions of *P. olivaris*, which can spread widely once a population is established, and highlight the importance of prevention and sensitive early detection methods to prevent the spread of *P. olivaris* in the future.

## 1 INTRODUCTION

Flathead catfish *Pylodictis olivaris* (Rafinesque 1818) is a large, predatory ictalurid species native to the Mobile, Rio Grande, and Mississippi river basins, including the Ohio River basin in western Pennsylvania. It has been widely introduced—both intentionally and unintentionally by state management agencies and illegally by anglers—into watersheds in the western United States, the Great Lakes drainages, and drainages along the Atlantic Slope from Florida to Pennsylvania (Brown et al., 2005; Dobbins et al., 2012; Fuller & Whelan, 2018; Jackson, 1999). Introduced *P. olivaris* populations can be established by a small number of individuals and undergo rapid population expansions (Dobbins et al., 2012; Granfors, 2014; Smith et al., 2021). For example, 11 *P. olivaris* released into the Cape Fear River, North Carolina, established a population that spread through 200 km of the river system and comprised most of the ecosystem's fish biomass just 13 years after introduction (Guier et al., 1984).

*P. olivaris* have life‐history and behavioral traits that influence their ability to successfully invade and spread in new environments. *P. olivaris* are long lived, with individuals reaching 25 years or older in some populations (Bodine et al., 2016; Schall & Lucchesi, 2021), but can reach sexual maturity quickly, with some fish maturing as early as age 2 in faster‐growing populations (Munger et al., 1994). Introduced populations often exhibit faster growth rates than native populations of *P. olivaris* and may have shorter generation times (Kwak et al., 2006; Sakaris et al., 2006). Female *P. olivaris* produce tens to hundreds of thousands of eggs during a breeding season (Colehour, 2009; Jackson, 1999), making them highly fecund, although there is limited information on how their reproductive ecology varies with ontogeny and among populations. In addition to longevity, early age at maturation, and high fecundity, *P. olivaris* are capable of long‐distance dispersal in large river ecosystems. Although they are usually sedentary, *P. olivaris* migrate between seasonal habitats, and typical distances for these seasonal migrations can vary from 3 to >20 km, with the furthest annual migrations often exceeding 100 km (Daugherty & Sutton, 2005; Travnichek, 2004; Vokoun & Rabeni, 2005). The greatest recorded distance for annual movements is 750 km (Garrett & Rabeni, 2011). Combined, these traits may allow small founding populations of *P. olivaris* to rapidly grow and spread in new systems when they are introduced.

*P. olivaris* were first detected in the Susquehanna River and Delaware River watersheds in 1991 and 1999, respectively, although there were no other confirmed observations of *P. olivaris* in the Susquehanna River until 2002 (Brown et al., 2005). Since then, *P. olivaris* populations have grown and expanded throughout reaches that were undammed or accessible through fish ladders in the Susquehanna, Juniata, Schuylkill, and Delaware rivers (Smith et al., 2021). The sources of *P. olivaris* introductions in these systems are not known; however, the current accepted mechanism of introduction is intentional introductions by anglers, but accidental stocking of fingerlings alongside intentionally stocked channel catfish and range expansion through the Chesapeake Bay from introduced populations in the Potomac River have not been ruled out (Brown et al., 2005).

Understanding introduction pathways and mechanisms of population expansion has implications for management and monitoring to protect ecosystems that may be at risk of nonnative species invasion (Abdelkrim et al., 2005; Novoa et al., 2020). However, because founding populations can consist of a small number of individuals, founding events and routes of invasion are often difficult to detect (Lodge et al., 2006). To that end, genetic analyses of invasive populations can be powerful tools to retroactively reconstruct the demographic history and patterns of connectivity of invasive populations, ultimately providing a means to assess the risk for future expansion and establishment of new populations (Le Roux & Wieczorek, 2009).

In this study, we utilized recently developed microsatellite markers for *P. olivaris* (White et al., 2021) for fine‐scale discrimination of population structure and demographic history modeling to understand invasion pathways for *P. olivaris* in the Susquehanna and Delaware river basins. We conducted a genetic survey of *P. olivaris* from the Susquehanna and Delaware river watersheds to (1) identify population structure and patterns of genetic differentiation within and among the introduced populations, (2) determine the phylogenetic relationships among introduced *P. olivaris* populations, and (3) estimate the timing and demographic parameters of the introduction of *P. olivaris* to these Pennsylvania watersheds.

## 2 METHODS

### 2.1 Sampling

**FIGURE 1:**
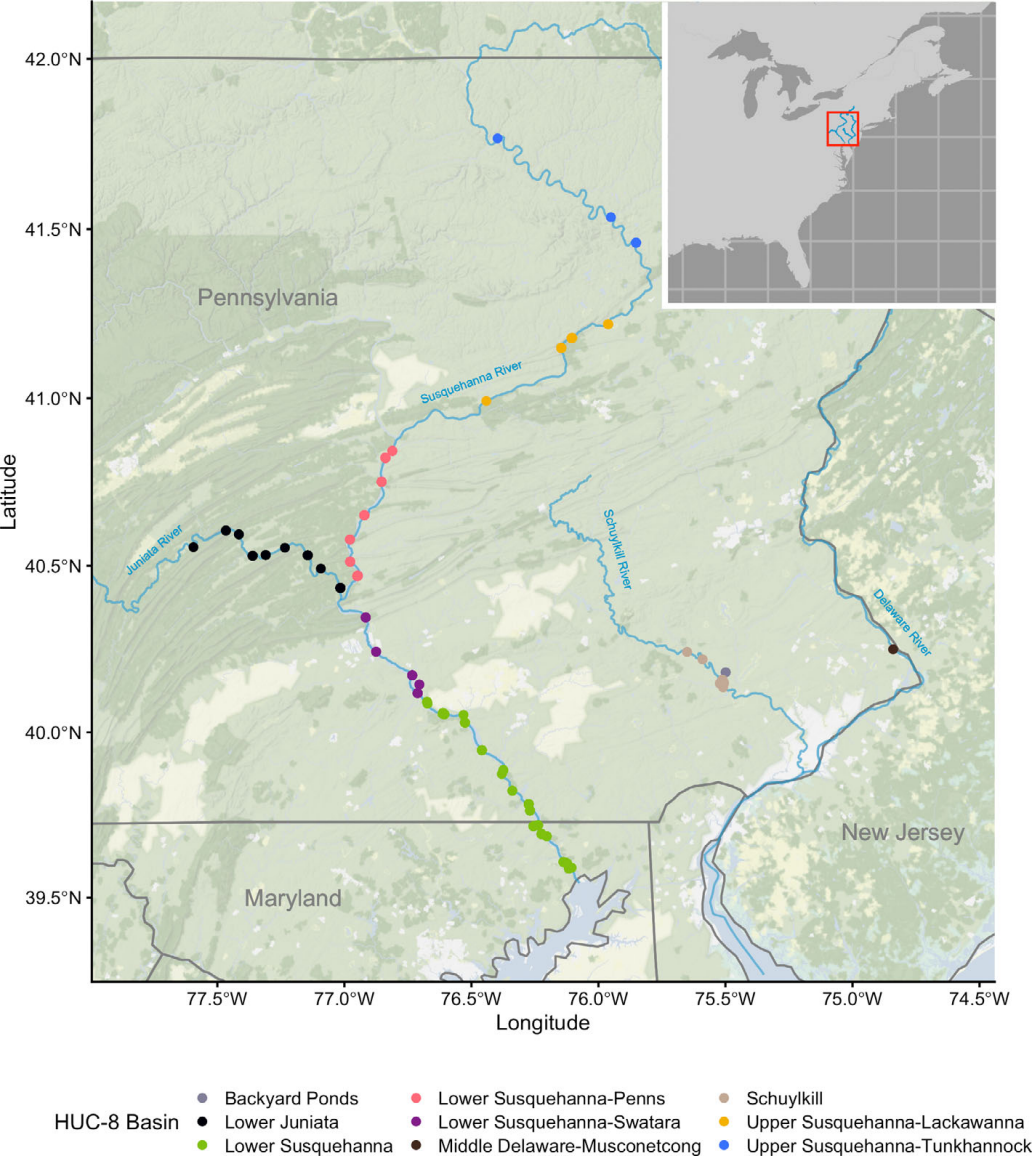
Map of the study area showing the locations of the sample sites color coded by Hydrological Unit Code (HUC) 8 level basins.

*P. olivaris* were captured from June to October 2020 and from April to October 2021 using low‐ and high‐frequency electrofishing (Bodine et al., 2013), baited tandem hoop nets, and angling, and from fish passage through a hydropower dam fish lift. Sampling was conducted in two river basins, the Susquehanna River basin, including the Susquehanna River from 18 to 391 km upstream of the river mouth and the Juniata River from the confluence with the Susquehanna River to 78 km upstream, and the Delaware River basin, including the Schuylkill River from 73 to 113 km upstream of the river mouth. In total, 655 *P. olivaris* genetic samples were collected from 45 unique sites (Figure 1), namely 5 sites in the Schuylkill River; a private pond ~1 km from the Schuylkill River in Collegeville, Pennsylvania (hereafter referred to as “Backyard Ponds”); 1 location in the Delaware River, and 38 sites from the Susquehanna River basin. Fish from the Susquehanna River basin were sampled by the Pennsylvania Fish and Boat Commission (*N* = 327), the Maryland Department of Natural Resources (*N* = 200), and the Susquehanna River Basin Commission (*N* = 56). Fish from the Schuylkill River (*N* = 41) and from a nearby private pond in Collegeville, Pennsylvania (*N* = 3), were collected by angling. After fish were captured, an ~1‐cm^2^ sample of the caudal fin was collected from each individual and preserved in 95% ethanol. Fish were all lethally sampled as part of other research projects or invasive species removal protocols; no fish could have been resampled. Sampling primarily targeted adult fish, ranging in size from 58 to 1250 mm (mean ± SD: 556 ± 216 mm). Sample sizes at each site ranged from *N* = 1 to 176 (median *N* = 6).

### 2.2 Ethics statement

All fish sampling and tissue collections were conducted in agreement with the Pennsylvania State University Institutional Animal Care and Use Committee (PROTO202102088 and PROTO201901210).

### 2.3 DNA extraction and microsatellite genotyping

Fin clips were extracted using DNEasy Blood and Tissue Kits (Qiagen). Individuals were genotyped on an ABI 3500 Genetic Analyzer at the U.S. Geological Survey Eastern Ecological Science Center using the panel of 13 microsatellite loci described in White et al. (2021). Individual genotypes were screened for missing data (Kamvar et al., 2014, 2015), and individuals with greater than 25% missing data (four or more loci) were removed. Microsatellite markers were also checked for null alleles using “PopGenReport” (Adamack & Gruber, 2014) in R (R Core Team, 2023). Markers that had null alleles with estimated frequencies significantly >0.05 were excluded from subsequent analyses unless otherwise noted. Linkage disequilibrium (LD) among the microsatellite markers was assessed using global and pair‐wise permutation tests of the multilocus approximation of the index of association, $\bar{r}D$ (Brown et al., 1980; Kamvar et al., 2014, 2015). Markers were also tested for deviations from Hardy–Weinberg equilibrium (HWE) using “hw.test” (Paradis, 2010). LD and HWE were evaluated for the overall dataset and separately for each river with more than one individual (Susquehanna, Juniata, and Schuylkill).

Prior to population genetic analysis, the samples were screened for sibling groups using COLONY (Jones & Wang, 2010). Although the practice of removing siblings from population genetics datasets can introduce additional biases in some situations (Waples & Anderson, 2017), it may be warranted for this dataset due to unbalanced sampling and the possibility for a few large families to be overrepresented at some sample sites. Individuals were considered part of a sibling group only if COLONY classified them as a full sibling with another individual from the same sample site with a probability >0.9. One member per sample site was randomly chosen among the individuals without any missing data from each group of full siblings to be kept in the final dataset.

### 2.4 Population genetic statistics and population structure

To calculate population genetic statistics, sample sites were organized by Hydrological Unit Code 8 (HUC‐8) level basins from the USGS Watershed Boundary Dataset (Figure 1) because these units grouped sites according to a standardized representation of hydrological connectivity and specifically allowed us to assess population genetic trends across downstream and upstream sections of the Susquehanna River. Samples collected from the Backyard Ponds in Collegeville, Pennsylvania, were categorized separately from the other samples collected from the Schuylkill River HUC‐8 basin. HUC‐8 level basins were analysed for private alleles (Kamvar et al., 2014, 2015), rarefied allelic richness based on the HUC‐8 basin with the smallest sample size (*N* = 7; Goudet & Jombart, 2022), expected and observed heterozygosity (Jombart, 2008), and the inbreeding coefficient *F*_IS_ (Keenan et al., 2013). Analysis of private alleles included markers with null alleles. Rarefied allelic richness and *F*_IS_ were not calculated for the Delaware River and Backyard Ponds due to small sample sizes (*N* = 1 and *N* = 3, respectively). Wilcoxon rank‐sum exact tests were used to determine if observed heterozygosity significantly diverged from expected heterozygosity within each HUC‐8 basin.

Pair‐wise *F*_ST_ was calculated among HUC‐8 basins and among sample sites using Weir and Cockerham's (1984) implementation (Goudet & Jombart, 2022). The proportion of the population differentiation explained by HUC‐8 basins and sample sites was tested using AMOVA (Excoiffer et al., 1992; Kamvar et al., 2014, 2015), and the statistical significance of these proportions was evaluated using permutation tests (Dray & Dufour, 2007; Thioulouse et al., 2018). Linearized *F*_ST_ values were also tested for patterns of isolation by distance in the Susquehanna and Schuylkill rivers separately using maximum‐likelihood population effect (MLPE) models, with pair‐wise river distances calculated using the R package “riverdist” (Tyers, 2022) and sample sites as random effects (Clarke et al. 2002; Peterman, 2018).

Clustering analyses were performed using Structure (Pritchard et al., 2000) and *k*‐means clustering using discriminant analysis of principal components (DAPC; Jombart, 2008). Structure runs were automated and parallelized using StrAuto (Chhatre & Emerson, 2017). An initial set of exploratory runs with five replicates per *K* of *K* = 2–40 with 50,000 burn‐in and 100,000 iterations was used to narrow the range of *K* clusters to explore in a more thorough set of Structure runs with 10 replicates per *K* of *K* = 2–8 with 500,000 burn‐in and 1 million iterations. The optimal number of clusters from Structure runs was determined using three methods: (1) maximum log likelihood averaged across runs for a given *K*, (2) Evanno's *∆K* method (Evanno et al., 2005), and (3) MedMeaK and the other supervised metrics described by Puechmaille (2016). The Puechmaille metrics threshold for assigning a subpopulation to a cluster was set to 0.5, meaning the mean or median assignment probability for a given subpopulation had to be >0.5 to be considered as belonging to a cluster. Two sets of the Puechmaille metrics were used, one that used HUC‐8 basins as a priori subpopulations and one that used the sample sites as a priori subpopulations. DAPC clusters were selected using the “diffNclust” and “goodfit” algorithms in the “find.Clusters” command from the “adegenet” package in R (Jombart, 2008) along with a visual inspection of the Bayesian information criterion curve for *K* = 1–40. The “optim.a.clust” function was used to select the number of principal components to retain while avoiding overfitting (Jombart, 2008). This process was repeated for samples exclusively from the Susquehanna River basin, but hierarchical Structure results were nearly identical to the analysis with the full dataset.

### 2.5 Demographic history analysis

Effective population size was estimated using the LD method in NeEstimator, version 2.1, with random mating and a minimum allele frequency of 0.05 (Do et al., 2014; Hill, 1981; Waples, 2006; Waples & Do, 2010). Samples were divided into three groups, the Schuylkill River (not including the Backyard Pond samples), the Lower Susquehanna HUC‐8 basin, and all other Susquehanna basins, including the Juniata River based on the highest‐order consensus clusters supported by Structure and DAPC analyses. Individuals with a total length <274 mm (expected length at age 2 in the Susquehanna River; Massie et al., 2018) were removed from the datasets input into NeEstimator to limit the effects of overlapping generations biasing estimation of effective population size (Moyer et al., 2012; Waples & Yokota, 2007).

To test for evidence of recent bottlenecks, *M*‐ratio tests were run on the same groups as described earlier using the “Critical_M” simulation script provided by Garza and Williamson (2001). The *M*‐ratio test is based on allele frequency patterns in microsatellites and other size‐varying genetic markers where populations undergoing bottlenecks tend to lose relatively rare alleles of intermediate size while the range in allele size typically shrinks at a slower relative rate. This leads to larger steps between alleles on average than in large, stable populations (Garza & Williamson, 2001). The *M*‐ratio is the number of alleles divided by the range in allele sizes, and the “Critical_M” script builds a distribution of *M*‐ratio values under equilibrium conditions and outputs *M*_C_, the bottom 95th quantile of the distribution under which there is statistically significant evidence of a genetic bottleneck (Garza & Williamson, 2001). To build this distribution, the script must be provided with parameters: theta equal to 4 * effective population size * mutation rate (*μ*); *p*_*s*_, the mean percentage of mutations that add or delete only one allele; and *∆*_*g*_, the mean size of larger mutations. A minimum theta (*θ*) was calculated for each population using the lower 95% CI for the estimate of effective population size calculated using NeEstimator, version 2.1, and a mutation rate of 1.52 × 10^−4^, the lower 95% CI of the rate estimated for microsatellites in the common carp (*Cyprinus carpio*; Yue et al., 2007), the closest relative of *P. olivaris* with an empirically estimated microsatellite mutation rate. *p*_*s*_ = 0.5477 and *∆*_*g*_ = 2.2105 were calculated from patterns in microsatellite frequencies from the whole dataset of genotyped *P. olivaris*. The *M*_C_ values calculated using the “Critical_M” script were then compared to the *M*‐ratio of each population.

The number of generations since founder events and founding population sizes were estimated for each population where significant evidence of a bottleneck was found using the “OnePopFounderFlush” model in MIGRAINE (Rousset et al., 2018). The “OnePopFounderFlush” model assumes a one‐time discrete change in population size from the ancestral population to the founding population, followed by a continuous increase in population size to the current population. The model was run using a generalized stepwise model (GSM) allowing for mutations larger than a single step. Five parameters were estimated by the model: (1) the current population size; (2) the founding population size; (3) the time in generations from the founding population to the current population; (4) the ancestral population size; and (5) *pGSM*, a parameter defining the frequency of mutations larger than a single step. For each population, an initial exploratory set of five MIGRAINE iterations were run with 500 points and 1000 runs per point. The parameter estimates from the exploratory set were used to define the parameter space to explore using a more thorough set of 10 MIGRAINE iterations with 2000 points and 5000 runs per point.

**FIGURE 2:**
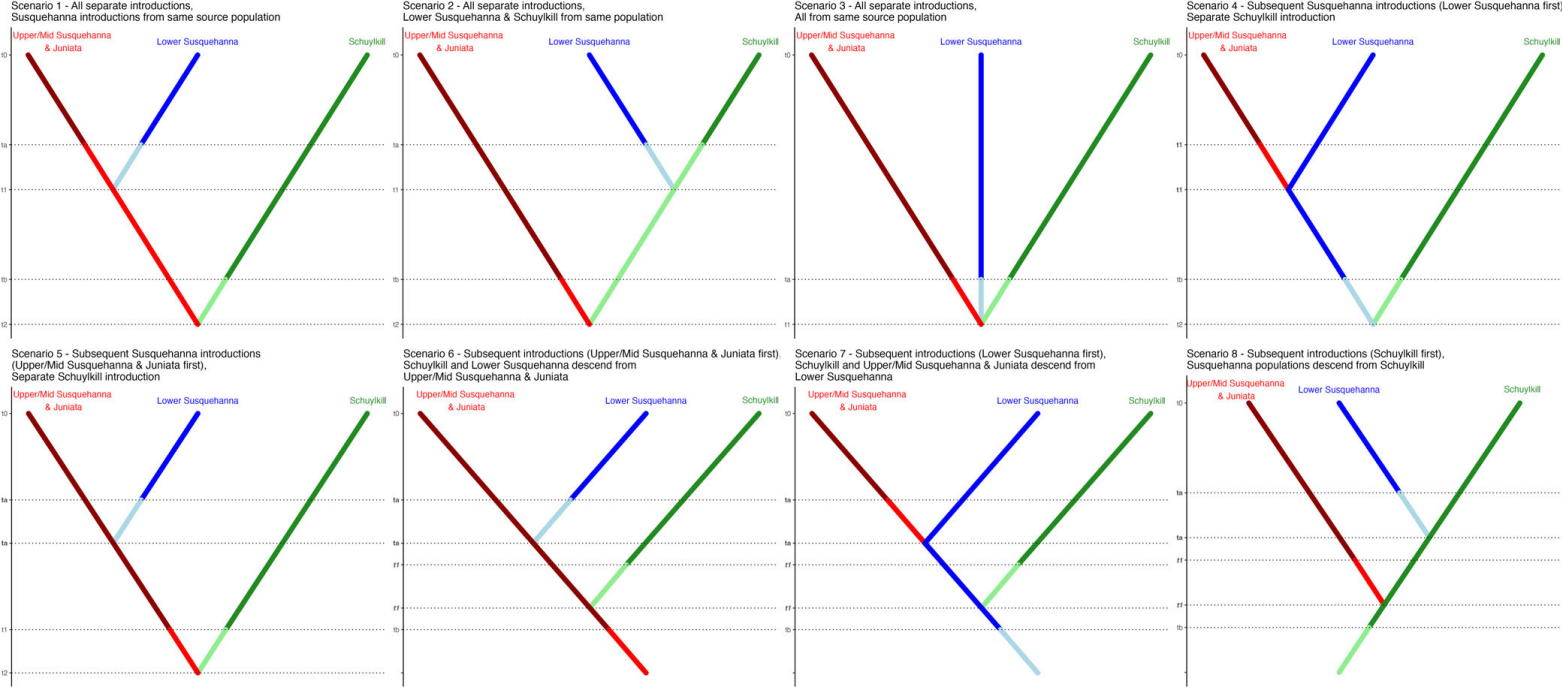
Visual representations of the eight scenarios compared in the diyABC analysis. In each scenario, t0 is the time when each population was sampled. Changes in population size are signified by lighter‐colored line segments in the dendrogram. Horizontal dotted lines represent time points where changes in population size or population differentiation occur. Note that in some scenarios, changes in population size and population differentiation occur at the same time point (labeled with the same time on the *y*‐axis) but are marked by separate horizontal dotted lines so the segments representing the changes in population size during founder events can be clearly seen.

To help determine whether invasive *P. olivaris* populations were founded separately or through consecutive founding events, a coalescence analysis was conducted using DIYABC Random Forest, version 1.0 (Collin et al., 2021). Eight scenarios were tested, varying the relative order of coalescence and discrete changes in population size meant to represent bottlenecks caused by founder events among the three populations (Schuylkill River, Lower Susquehanna River, and the Middle/Upper Susquehanna and Juniata rivers; Figure 2). Scenarios 1–3 modeled separate introductions for each population, allowing bottlenecks to occur separate from coalescence among the three lineages. Scenarios 4 and 5 allowed separate introductions in the Schuylkill and Susquehanna rivers but forced one population within the Susquehanna River to be founded by the other by linking the discrete population change founding event and coalescence of the Susquehanna lineages to the same time step. Scenarios 6–8 modeled situations where all the populations descended from a single introduction, and each coalescence time was also linked to a bottleneck event. In scenarios 1 and 4–7 the Lower Susquehanna and the Middle/Upper Susquehanna and Juniata populations are more closely related to each other than the Schuylkill, whereas in scenarios 2 and 8, one of the Susquehanna populations is more closely related to the Schuylkill population than the other Susquehanna population. The training set of coalescent demographic models was generated using 1 million replicates. In the DIYABC replicates, simulated population sizes were drawn from uniform distributions from 4 to 10,000, time steps with bottleneck founder events were drawn from uniform distributions of 1–200 generations before time 0, and ancestral coalescence events (those not tied to an introduction in our study area) were drawn from uniform distributions of 1–5000 generations before time 0. The limits on these distributions were informed by the estimates generated by the MIGRAINE analysis. Simulated mutation rates were drawn from a uniform distribution based on the 95% CI of the common carp mutation rate (1.52 × 10^−4^–1.63 × 10^−3^ mutations/locus/generation; Yue et al., 2007). Scenarios were compared to the observed dataset, with 10,000 random forest trees assessed using 39 within‐ and between‐population summary statistics (Beaumont, 2008; Garza & Williamson, 2001; Miller et al., 2005). Estimated parameter values and CIs were obtained from the best‐performing scenarios using 40,000 random forest trees. Data used in this analysis are available on ScienceBase (10.5066/P13QEOK3), and the code for model fitting is available on GitLab (10.5066/P13VRDLF).

## 3 RESULTS

### 3.1 Genotype filtering

Microsatellite loci varied from two to eight alleles per locus (mean = 4.85, median = 5). The global rate of missing data prior to filtering was 0.52%. Two individuals were missing genotypes at four or more loci and were removed from the dataset. Two loci, *IpCG00189* and *Ip271*, showed evidence of null alleles. The estimated null allele frequency of *IpCG00189* was relatively low (95% CI: 0.03–0.08) and was unlikely to significantly affect results, whereas the null allele frequency for *Ip271* was much higher (95% CI: 0.147–0.216) and was removed from subsequent analyses unless otherwise noted. There was significant global LD detected among the microsatellite markers in the full dataset and in the Susquehanna and Schuylkill rivers (all *p* = 0.001), but not in the Juniata River (*p* = 0.075). Pair‐wise testing of LD also did not reveal any pairs of markers that significantly deviated from linkage equilibrium consistently across rivers, so no loci were removed due to LD. Similarly, HWE tests found significant global deviations from HWE expectations (*p* = 0.001), and one marker, *Ip271* again, exhibited systematic deviation across all rivers, likely due to the prevalence of null alleles. When *Ip271* was removed from the dataset, significant LD detected had $\bar{r}D$ values <0.05 and was unlikely to affect subsequent analysis, and only loci in the Susquehanna River significantly deviated from the HWE. When the Susquehanna samples were divided by HUC‐8 basin, no loci significantly deviated from the HWE in more than one HUC‐8 basin, indicating that deviations in the river were likely being driven by spatial population structure.

COLONY identified 63 sibling groups encompassing 185 individuals. One individual per family group per sample site was retained. A total of 79 putative siblings were filtered out of the dataset, nearly half (37, 46.8%) of which belonged to two large family groups (>10 individuals) found in two different sample sites in the Upper Susquehanna–Lackawanna basin. The final dataset included *N* = 574 individuals from the Susquehanna River (*N* = 476), Juniata River (*N* = 61), Schuylkill River (*N* = 33), Backyard Ponds (*N* = 3), and the Delaware River (*N* = 1; Table 1).

**TABLE 1:** Sample size after filtering (*N*) and population genetic statistics for each HUC‐8 basin.

Basin	*N*	Private alleles	Ar	*H*_obs_	*H*_exp_	*F*_IS_ (95% CI)
Delaware River	1	2	–	0.667	0.333	–
Backyard Ponds	3	0	–	0.639	0.463	–
Schuylkill	33	3	3.101	0.504	0.507	−0.016 (−0.234 to 0.198)
LS	263	8	2.629	0.499	0.510	0.019 (−0.076 to 0.114)
LS–Penns	119	0	2.458	0.502	0.490	−0.025 (−0.163 to 0.116)
LS–Swatara	39	0	2.514	0.512	0.512	−0.066 (−0.300 to 0.174)
Juniata	61	0	2.423	0.520	0.484	−0.083 (−0.272 to 0.111)
US–Lackawanna	48	0	2.609	0.562	0.518	−0.088 (−0.295 to 0.119)
US–Tunkhannock	7	0	2.500	0.512	0.501	0.022 (−0.486 to 0.424)

*Note*: Population genetic statistics include the number of private alleles, rarefied allelic richness (Ar), observed heterozygosity (*H*_obs_), expected heterozygosity (*H*_exp_), and the inbreeding coefficient (*F*_IS_).

Abbreviations: LS, Lower Susquehanna River; US, Upper Susquehanna River.

### 3.2 Basic population genetic statistics

The Lower Susquehanna HUC‐8 basin had the highest number of private alleles (*N* = 8), followed by the Schuylkill River (N = 3), and the single fish collected from the Delaware River (*N* = 1; Table 1). Allelic richness did not vary much among basins, ranging from 2.42 in the Juniata River to 3.10 in the Schuylkill River (Table 1). Similarly, observed and expected heterozygosities were close to 0.5 for all basins and showed no patterns of either heterozygote excess or deficiency (all Wilcoxon rank‐sum exact tests *p* > 0.05; Table 1). Inbreeding coefficients were not significantly different from 0 for all HUC‐8 basins (Table 1).

### 3.3 Population structure

**FIGURE 3:**
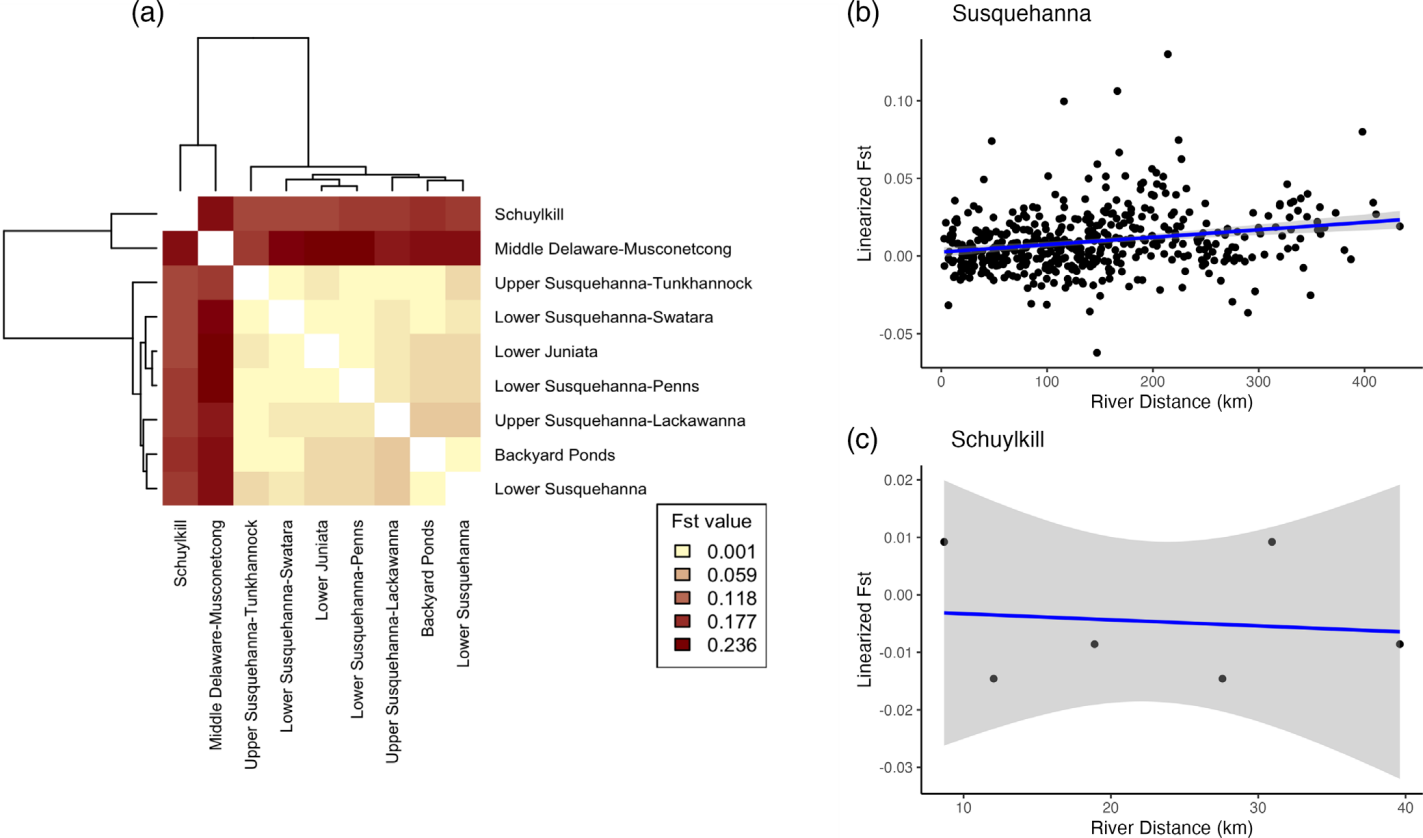
Visualizations of pair‐wise *F*_ST_ among sampled flathead catfish. Pair‐wise *F*_ST_ values among basins are represented as a heat map, with (a) the corresponding dendrogram showing a strong division between the Susquehanna River basin and the Delaware/Schuylkill River basin. Pair‐wise *F*_ST_ values among sample sites are plotted against river distance to show the pattern of isolation by distance (IBD) within (b) the Susquehanna and Juniata rivers and lack of an IBD pattern in (c) the Schuylkill River.

AMOVA revealed small, but statistically significant, proportions of genetic variation could be explained by river HUC‐8 basins (7.39%, *p* = 0.001) and by sample sites (1.01%, *p* = 0.012). Between‐river *F*_ST_ values for HUC‐8 basins were highest between the Susquehanna HUC‐8 basins and the Delaware River fish (*F*_ST_: 0.209–0.256) and were slightly lower between Susquehanna basins and the Schuylkill basin (*F*_ST_: 0.155–0.180; Figure 3a). Notably, fish from the Backyard Ponds near the Schuylkill River were similarly differentiated from the *P. olivaris* collected from the Schuylkill (*F*_ST_: 0.161) and grouped with the Susquehanna River basins (*F*_ST_: −0.038 to 0.010). *F*_ST_ values between basins within the Susquehanna River showed little genetic differentiation (*F*_ST_: 0.002–0.033; Figure 3a).

Within the Susquehanna River, the MLPE models, including effects of isolation by distance, outperformed the null model with population random effects only (∆AIC = 6.00; Figure 3b) and indicated a positive relationship between river distance and genetic distance. The MLPE model with river distances was also better supported than the null model in the Schuylkill River (∆AIC = 5.25), but river distance had an inverse relationship with *F*_ST_ (Figure 3c).

**FIGURE 4:**
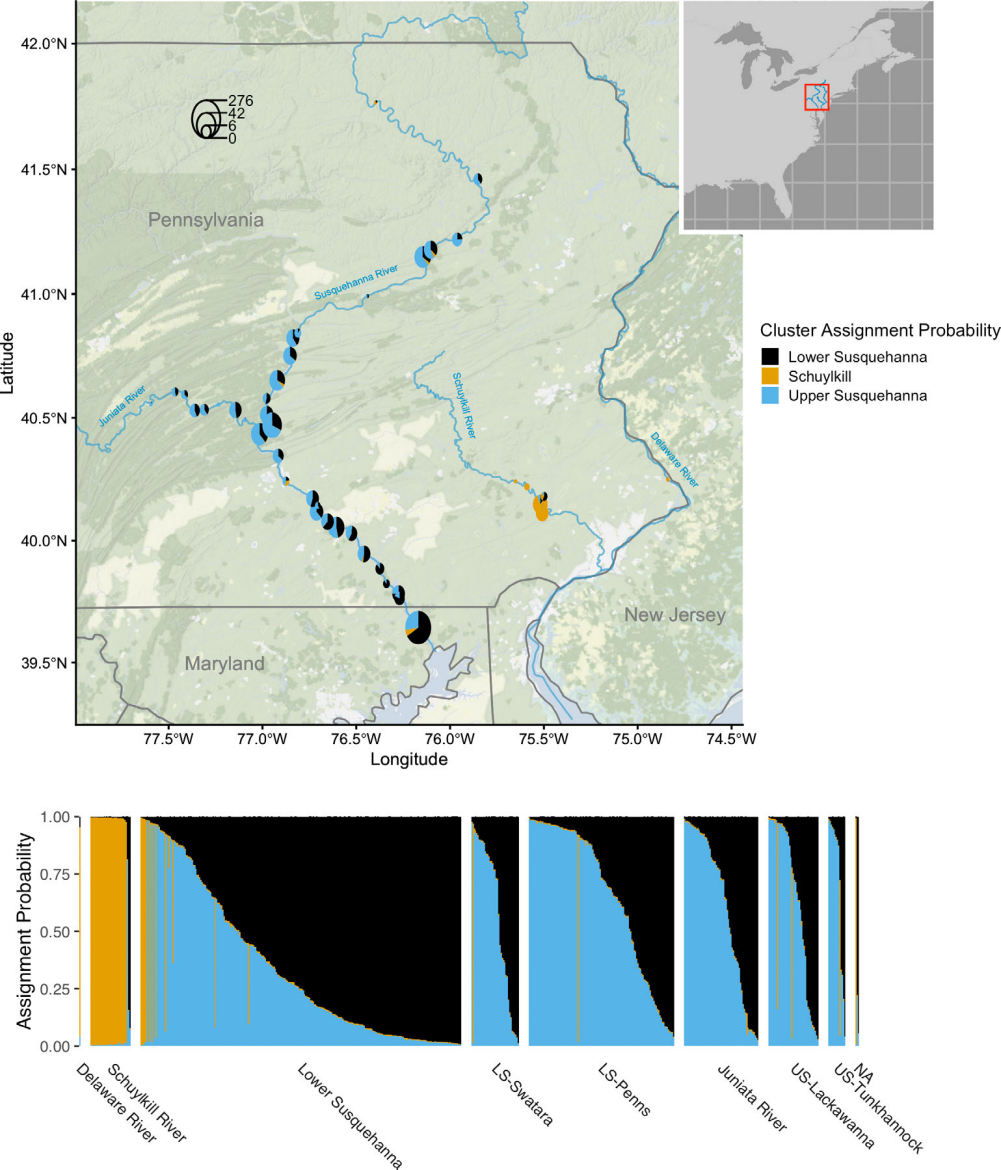
Results of the structure clustering analysis for *K* = 3 subpopulations. Aggregated assignment probabilities within sample sites are plotted as pie plots on a map of the study area to show the geographic pattern of population structure (top), and individual assignment probabilities organized by basin are shown as a bar plot (bottom). A distinct subpopulation is found primarily in the Schuylkill River (orange), and two subpopulations are widely distributed throughout the Susquehanna River basin, one found more commonly in the Lower Susquehanna (black) and the other found more commonly in the Upper Susquehanna (blue).

The metrics used to evaluate the optimal number of clusters in the Structure analysis disagreed. Evanno's ∆*K* method identified *K* = 2 as the optimal number of clusters, splitting the fish from the Delaware River and the Schuylkill River apart from the Susquehanna River basin (Figure S1a). The Puechmaille (2016) metrics with HUC‐8 basins as a priori subpopulations agreed at *K* = 3 (Table S1), further splitting the Susquehanna River into two widespread admixed clusters, one more common in the Lower Susquehanna and one more common in the Middle/Upper Susquehanna and Juniata rivers (Figure 4). Maximum log likelihood and the Puechmaille metrics using sample sites as a priori subpopulations agreed at *K* = 6 (Figure S1b; Table S2), splitting the Susquehanna into five widely admixed clusters and assigning nearly all individuals collected from the Schuylkill to one unique cluster (Figure S2).

DAPCs identified *K* = 5 and *K* = 9 as the optimal number of clusters based on the “diffNclust” and the “goodfit” algorithms, respectively. In both cases, the Schuylkill River fish were primarily assigned to one cluster, whereas the remaining clusters divided groups in the Susquehanna River with some weak spatial organization (Figures S3 and S4).

### 3.4 Demographic analyses

Contemporary effective population sizes estimated using NeEstimator, version 2.1, were largest for the Lower Susquehanna River population, followed by the other Susquehanna River HUC‐8 basins, whereas the Schuylkill River population produced the smallest Ne estimate (Table 2). All three populations showed strong signals of population bottlenecks according to the *M*‐ratio tests, as all the observed *M* values were much lower than the *M*_C_ values produced by the “M_Critical” simulations (Table 3).

**TABLE 2:** Contemporary effective population sizes (Ne) calculated for three population groups of invasive flathead catfish in the Schuylkill River, Lower Susquehanna, and all other Susquehanna River sites as estimated using the linkage disequilibrium method in NeEstimator, version 2.1.

Population	Ne estimate	95% CI (parametric)	95% CI (jackknife)
Schuylkill River	9.0	6.0–12.5	3.6–17.4
Lower Susquehanna River	164.2	107.2–283.6	87.2–426.1
Middle/Upper Susquehanna and Juniata rivers	42.7	32.0–56.7	29.0–62.3

*Note*: Ne point estimates are shown with both the parametric and jackknife 95% CIs provided by NeEstimator.

**TABLE 3:** Results of *M*‐ratio tests comparing the observed *M* value to the critical *M* of simulated *M* values under equilibrium conditions for the three populations.

Population	Observed *M*	Critical *M*
Schuylkill River	0.291	0.708
Lower Susquehanna River	0.321	0.710
Middle/Upper Susquehanna and Juniata rivers	0.281	0.708

*Note*: Observed *M* values lower than the critical *M* value indicate statistically significant evidence of recent population bottlenecks.

The “OnePopFounderFlush” MIGRAINE models for the two Susquehanna River populations resolved around similar parameter estimates with stable likelihood surfaces. The Lower Susquehanna was estimated to be founded by 4.6–131 individuals 11–398 generations ago, with the estimates being slightly lower for the population in the Middle/Upper Susquehanna and Juniata rivers (Table 4; Table S3). For the Schuylkill River, the demographic model did not resolve as easily, and likelihood surfaces were more variable. Additionally, the results for the Schuylkill River do not converge on reasonable values for some parameters using the assumed microsatellite mutation rate (Table 4).

**TABLE 4:** Parameter estimates and CIs for the “OnePopFounderFlush” model in MIGRAINE for each population.

Population	Current population size	Generations since founding event	Founding population size	Ancestral population size	pGSM
Schuylkill River	67.0 [24.1–288.2]	0.001 [0.0003–0.0025]	0.11 [0.07–0.23]	513.9 [478.4–535.1]	0.619 [0.615–0.702]
Lower Susquehanna River	464.9 [192.9–1310.7]	59.0 [11.1–398.0]	17.5 [4.6–131.7]	1005.4 [355.2–3551.3]	0.537 [0.378–0.662]
Middle/Upper Susquehanna and Juniata rivers	389.8 [94.9–2454.6]	19.6 [1.4–299.4]	3.2 [0.7–44.4]	1882.6 [457.3–7468.5]	0.397 [0.200–0.612]

*Notes*: pGSM is a parameter that defines the rate of mutations where the microsatellite changes more than one step (the length of the repeated motif) in the generalized stepwise mutation model implemented by MIGRAINE. Parameter estimates have been rescaled from the standard MIGRAINE output using a mutation rate of 5.56 × 10^−4^ mutations/locus/generation (Yue et al., 2007) to more easily interpretable values. Raw MIGRAINE estimates are provided in Table S3.

Abbreviation: GSM, generalized stepwise model.

In the DIYABC Random Forest analysis, scenario 6 was the best‐performing model with a posterior probability of 0.513 and being the top scenario in 3079 of the 10,000 random forest trees. Scenarios 1, 4, 5, and 7 also performed well, being selected by 2019, 1561, 1776, and 1312 of the 10,000 random forest trees, respectively. Scenarios 1, 4, and 5 all modeled very recent divergence between the two populations in the Susquehanna River and allowed longer divergence times between the Susquehanna River populations and the Schuylkill River population. Scenario 1 modeled a divergence between the two Susquehanna clusters prior to their introduction into the Susquehanna River (i.e., the Middle/Upper and Lower Susquehanna River clusters were founded by separate founders from the same source population). Scenario 4 modeled a situation where the Lower Susquehanna population was founded first and the other Susquehanna River population diverged from the established invasive population in a second founder event at a later time, and scenario 5 modeled a similar situation except the Middle/Upper Susquehanna and Juniata populations were founded first (Figure 2; Table 5). Scenarios 6 and 7 modeled situations where the Schuylkill River population directly descended from either the Middle/Upper or Lower Susquehanna populations, respectively (Figure 2; Table 5). The observed data were most similar to marginal outliers of the simulated datasets along linear discriminant axes (LDA) 1 and 2 (Figure S5a) but were well represented across the best‐performing scenarios along the other LDAs (Figure S5b,c). Additionally, there was a high amount of confusion among all scenarios, with a global error rate of 0.440. Scenarios 1, 4, and 5 were most commonly confused with each other, and scenario 6 was most often confused with scenario 7, though there was relatively low confusion among these two groups (Table S4).

**TABLE 5:** Parameter estimates and 90% CIs for the diyABC Random Forest analysis for the best‐performing scenarios 1, 4, 5, and 6.

Scenario	N1 (Mid/Upper Susquehanna and Juniata)	N2 (Lower Susquehanna)	N3 (Schuylkill)	ta	t1
1 (All separate introductions, Susquehanna populations from same source)	3387 [298–8797]	5000 [725–9406]	4288 [476–9437]	48.5 [4–120]	98.4 [36–169]
4 (Subsequent Susquehanna introductions, mid/upper first, separate Schuylkill introduction)	1905 [288–6325]	4900 [1037–9557]	3187 [400–9173]	–	79.5 [23–161]
5 (Subsequent Susquehanna introductions, lower first, separate Schuylkill introduction)	1292 [192–4110]	5281 [1018–9563]	3904 [384–9228]	69.1 [16–143]	139.6 [59–196]
6 (All subsequent introductions, descended from mid/upper Susquehanna)	277 [75–600]	4829 [699–9547]	2322 [138–8625]	17.6 [4–43]	103.6 [40–169]

Scenario	tb	t2	Nsource1	Nsource2	Nsource3
1 (All separate introductions, Susquehanna populations from same source)	143.4 [73–195]	2156 [717–4014]	3413 [1158–6581]	5116 [874–9523]	5574 [1454–9712]
4 (Subsequent Susquehanna introductions, mid/upper first, separate Schuylkill introduction)	130.0 [47–194]	1835 [566–3887]	1813 [0–8554]	3035 [154–8918]	4401 [1572–8208]
5 (Subsequent Susquehanna introductions, lower first, separate Schuylkill introduction)	–	2221 [615–4258]	2535 [0–8636]	4196 [1538–7821]	5124 [514–9597]
6 (All subsequent introductions, descended from mid/upper Susquehanna)	151.9 [78–197]	–	2988 [0–9239]	5391 [1544–9455]	4891 [503–9514]

*Note*: All times are represented in generations.

## 4 DISCUSSION

Invasive *P. olivaris* population in the Mid‐Atlantic region is primarily genetically structured by river basins, likely due to separate founder events. Consistent with the timeline of suspected founder events based on historical detections, these populations have undergone recent bottlenecks, suggesting establishment slightly before the earliest detections. The genetic diversity of these introduced populations was low compared to similar species (e.g., wild channel catfish [*H*_obs_ = 0.76–0.79, *A* = 7.44–9, Janzen & Blouin‐Demers, 2023; *H*_obs_ = 0.65–0.71, *A* = 5.42–6.51, Sotola, 2016]; farmed channel and blue catfishes [*A* = 2.75–4.12, Lamkom et al., 2008; *H*_obs_ = 0.57–0.77, Perales‐Flores et al., 2007]), although there were no signs of excessive inbreeding despite the founder effects and low diversity. Genetic bottlenecks from founder events are commonly seen in introduced populations, but reductions in genetic diversity do not necessarily limit population growth and expansion (Bernos et al., 2023; Purcell & Stockwell, 2015; Yue et al., 2010). Measures of genetic diversity, coalescence analysis, and weak within‐basin genetic structure support a single founding event in the Susquehanna River followed by population expansion. There was likely a second separate founding event that established the Schuylkill River population, but it remains uncertain as to whether the Schuylkill River population was founded by fish from the Susquehanna River or another unsampled population. This study demonstrates the utility of genetic surveys of introduced populations to supplement historical records to enhance our understanding of the establishment and spread of invasive species.

### 4.1 Demographic reconstruction supports strong, recent population bottlenecks

Population bottlenecks are expected to be part of the demographic history for many populations of introduced species that are commonly founded by a small number of individuals (Barrett & Richardson, 1986; Dlugosch & Parker, 2008). Evidence for population bottlenecks was found for all *P. olivaris* in the Susquehanna and Schuylkill rivers. In the Susquehanna River subpopulations, there is a large amount of overlap in the estimated time of founding events, and due to the high degree of gene flow in the Susquehanna River, we cannot be certain whether the estimates are for the same or different founding events. Regardless, the estimated ranges for the timing of founding events overlap with historical observations of *P. olivaris* in the Susquehanna River, with the first records in the mainstem Lower Susquehanna near Safe Harbor Dam in 2002 (Brown et al., 2005), and the range of the introduced population subsequently expanding out from this site (Smith et al., 2021). Some populations of *P. olivaris* appear to mature relatively quickly, in as short as 2 years (Munger et al., 1994), and this would place the lower end of the 95% CI for the number of generations since the founder event in the Lower Susquehanna (11.1 generations) about the same time as the first recorded observation in 2002. The detection date of 2002 represents a minimum age for the introduced population, and both the MIGRAINE and DIYABC models suggest that the Susquehanna population could have been established several generations prior to the detection date.

There was also strong evidence for recent bottlenecks in the Schuylkill River, though it was more difficult to estimate the time since the founding event. This may reflect the relatively small sample size for the Schuylkill River (*N* = 33) compared to the Lower Susquehanna (*N* = 263) and the Middle/Upper Susquehanna and Juniata (*N* = 274). Additionally, if the Schuylkill River population was founded by individuals from another introduced population with a strong recent bottleneck, it may be difficult to calculate accurate estimates using the MIGRAINE model, which includes only one discrete decline in population size, when there are signals of multiple successive bottlenecks in the data.

These population bottlenecks have likely resulted in reduced genetic diversity, though genotype data from potential source populations would be necessary to confirm this. Evidence of bottlenecks comes primarily from low allelic richness and the *M*‐ratio prevalence of “missing” alleles, size gaps between alleles larger than one repeating motif of the microsatellite. There was no evidence for inbreeding nor any signs of heterozygote deficiency or excess, indicating that founder effects do not seem to have had any lasting effects on population vital rates. This pattern is somewhat counterintuitive but a common finding in many successful nonnative species (Purcell & Stockwell, 2015; Zhao et al., 2013).

### 4.2 Genetic differentiation in different watersheds: Evidence for separate introductions or subsequent founder events?

One of the most robust patterns in this study was the clear genetic differentiation of *P. olivaris* found in the Susquehanna and Schuylkill river systems. A unique genetic subpopulation in the Schuylkill River is supported across all levels of *K* in both Structure and DAPC analyses. Additionally, the Schuylkill River collection (excluding the single sample from the Delaware River) has the highest pair‐wise *F*_ST_ values compared to samples from HUC‐8 basins from the Susquehanna River (0.1463–0.1667). Despite the small sample size, *P. olivaris* in the Delaware River may also be differentiated from the Schuylkill and Susquehanna rivers as evidenced by high between‐HUC‐8 basin *F*_ST_ values (*F*_ST_: 0.1667–0.2209), and two private alleles unique to the Delaware River sample. More samples are necessary to determine whether this individual is representative of *P. olivaris* in the Delaware River, but, if so, it could suggest that there was another separate introduction that established *P. olivaris* in the Delaware River.

Although the Susquehanna River and Schuylkill River *P. olivaris* populations are clearly differentiated, there is conflicting evidence regarding the origins of the Schuylkill River population—was it established by fish transferred from the Susquehanna River or from another source? The most supported DIYABC scenario (scenario 6) suggests that the Schuylkill River population originated from the Susquehanna River. However, this model also suggests that the Susquehanna and Schuylkill river populations last shared ancestry at least 78 generations ago, long before records of *P. olivaris* appeared in either system. The other best‐performing DIYABC scenarios (scenarios 1, 4, and 5) model a separate introduction of *P. olivaris* to the Schuylkill River. Additionally, the presence of several private alleles in the Schuylkill River would also support scenarios with separate introduction events in each river. Some of the uncertainty surrounding the origins of introductions to the Susquehanna and Schuylkill rivers could be resolved with genotypes of *P. olivaris* from other native and older introduced populations that could have served as the source(s).

The population structure present in the Susquehanna and Schuylkill rivers indicates that both populations were founded from single sources. Unlike introduced populations with inputs from multiple sources, the *P. olivaris* in these rivers do not exhibit signs of excess heterozygosity that can result from contact between two or more previously isolated source populations, nor are there strong patterns of local genetic structure within sample areas (Castagné et al., 2023; Dlugosch & Parker, 2008; Zalewski et al., 2010).

### 4.3 Natural long‐distance and human‐mediated dispersal could explain weak spatial population structuring within watersheds

Although *P. olivaris* from the Susquehanna, Schuylkill, and Delaware rivers were clearly genetically differentiated from one another, little population structure was detected within collections from any single river. Pair‐wise *F*_ST_ values between HUC‐8 basins within each river were low, with maximum *F*_ST_ values near 0.03 between the northernmost upstream Upper Susquehanna–Lackawanna and Tunkhannock watersheds and the Lower Susquehanna watershed near the river mouth. Despite disagreement over the optimal number of clusters using different metrics for Structure and DAPC analyses, all clustering methods produced similar patterns, showing high admixture and little spatial organization throughout the Susquehanna River. This pattern is consistent with a continuous gradient of changing allele frequencies caused by isolation by distance, as statistical clustering methods often disagree on selecting discrete clusters when the underlying pattern is a continuous change and the samples are spread throughout the study area, as is the case in this study (Guillot et al., 2009; Meirmans, 2012).

The lack of a strong population structure in the Susquehanna River suggests that there are a few barriers restricting dispersal of *P. olivaris*. High connectivity within the Susquehanna River system may be the primary explanation for the rapid spread of *P. olivaris* from the site of initial introduction. Some *P. olivaris* occupying large rivers are known to disperse long distances (commonly >20 km, but up to 750 km; Brundage III & Park, 2022; Garrett & Rabeni, 2011; Travnichek, 2004). This long‐distance dispersal ability and recent introduction to the system could explain the low degree of population structure. Additionally, high connectivity may have been an important factor that allowed the initial introduction of *P. olivaris* to overcome negative consequences of initial founder effects. High gene flow throughout the introduced range would prevent the further loss of genetic diversity, as well as allow adaptive genetic variation to spread rapidly (Tepolt et al., 2022).

There is also evidence for some limited connectivity between watersheds that may be through natural dispersal, human‐mediated movement, or both. Nine putative migrants assigned to the Schuylkill River genetic subpopulation and two putative F1 offspring of Schuylkill River migrants were found in the Lower Susquehanna watershed in Maryland, and three possibly admixed individuals with both Susquehanna and Schuylkill river ancestry were found in the Upper Susquehanna–Lackawanna watershed (Figure 4). The tidal river mouths of the Susquehanna and Delaware river watersheds (including the Schuylkill River) are connected by the Chesapeake and Delaware (C & D) Canal. *P. olivaris* can tolerate salinity up to 14.5 ppt, and it has been speculated that the brackish water in the C & D Canal and adjacent tidal river mouths would not be a strict barrier to dispersal (Bringolf et al., 2005; Brown et al., 2005; Smith et al., 2021). Alternatively, people may be moving *P. olivaris* across watershed boundaries. This is highly likely to be the case for the three fish sampled from a private pond near the Schuylkill River. Two of the three individuals were assigned to Susquehanna River genetic subpopulations, and one was assigned to the Schuylkill River population.

## 5 CONCLUSIONS

This genetic survey of introduced *P. olivaris* found population structure primarily reflected spatial organization by major watersheds. The patterns of genetic diversity match expectations under scenarios where these populations were established by separate founder events from single sources. Genetic information from potential source populations would be useful to provide a more robust assessment of their introduction history (e.g., whether these populations descended from the same source population or different sources, and what was the most likely route of introduction). Lack of genetic data from potential source populations is a common limitation when studying invasive species (Le Roux & Wieczorek, 2009; Muirhead et al., 2008; Resh et al., 2021), and the microsatellite markers used in this study system present an opportunity to investigate important questions regarding the management of *P. olivaris* throughout its native and introduced range. Within watersheds, population structure was relatively weak, indicating high levels of gene flow throughout these large systems consistent with long‐distance dispersal behaviors of *P. olivaris* observed in other river systems. This high level of connectivity was found despite the presence of major dams on the Susquehanna River, suggesting there are a few barriers preventing movement of *P. olivaris* in these systems. *P. olivaris* have been observed using fish passage structures (Smith et al., 2021), and our results suggest that these obstacles do not noticeably restrict gene flow. Additionally, given the relatively few numbers of founders estimated by genetic analyses, small‐scale introductions (<20 individuals) may be sufficient to establish *P. olivaris* populations in new systems. This highlights the need for prevention and early detection if managers hope to contain introductions of *P. olivaris* in other systems in the future, and any management efforts aimed at reduction or removal of invasive populations are likely to be more effective if connectivity is considered.

*P. olivaris* in the Susquehanna and Delaware river watersheds exhibited signs of strong, recent bottlenecks and low genetic diversity. However, despite founder effects, these introduced populations do not show signs of elevated inbreeding. The high degree of connectivity and large number of breeding individuals would facilitate further range expansion without a high risk of greater inbreeding or a decline in the current genetic diversity. This adds to a growing body of literature showing that invasive populations often exhibit little genetic degradation after the initial founder effects when they are established (Bernos et al., 2023; Díez‐del‐Molino et al., 2013; Wellband et al., 2018). Additionally, the estimated small size of the founding populations highlights the invasion risk posed by a relatively few individuals and the need for intensive monitoring of at‐risk ecosystems if managers wish to detect introduced populations before they become established.

This study can be used by managers and stakeholders to understand the establishment history of *P. olivaris* in the Susquehanna and Delaware river watersheds and how *P. olivaris* has spread throughout these systems. Future genetic work on *P. olivaris* may focus on environmental factors influencing dispersal patterns to identify barriers or key migration corridors that could be used to predict the risk of future range expansion in different habitats. Additionally, genetic surveys throughout the native and introduced range of *P. olivaris* could be used to identify source populations, reconstruct invasion histories, and reveal patterns of genetic diversity that may be associated with successful establishment and expansion of introduced populations.

## AUTHOR CONTRIBUTIONS

Justin M. Waraniak conducted statistical analyses and wrote the manuscript with input from the other authors. Michael S. Eackles, Jason Keagy, David C. Kazyak, and Tyler Wagner secured funding for this work. Michael S. Eackles, Jason Keagy, Geoffrey D. Smith, Megan Schall, Shannon L. White, David C. Kazyak, and Tyler Wagner developed the study. Michael S. Eackles, Sydney Stark, Shannon L. White, Geoffrey D. Smith, and David C. Kazyak planned and managed fieldwork to collect genetic tissue samples. Shannon L. White conducted laboratory work and genotyping.

## FUNDING INFORMATION

This work was funded by the US Geological Survey. Jason Keagy was supported by the USDA National Institute of Food and Agriculture Federal Appropriations under project PEN04768 and accession number 1026660.

## Supporting information


**Figure S1.** Heuristics used to select the optimal number of *K* clusters from the Structure and DAPC (discriminant analysis of principal component) analyses, including the mean log likelihood along with SD across 10 independent runs of (a) Structure, (b) Evanno's ∆*K* measure, and (c) the Bayesian information criterion for *k*‐means clustering. The optimal number(s) of *K* are represented by vertical red dotted lines for each heuristic.

**Figure S2.** Results of the Structure clustering analysis for *K* = 6 subpopulations. Aggregated assignment probabilities within sample sites are plotted as pie plots on a map of the study area to show the geographic pattern of population structure (top), and individual assignment probabilities organized by basin are shown as a bar plot (bottom). A distinct subpopulation is found primarily in the Schuylkill River (orange), and five subpopulations are widely distributed throughout the Susquehanna River basin.

**Figure S3.** Results of the DAPC (discriminant analysis of principal component) clustering analysis for *K* = 5 subpopulations. Aggregated assignment probabilities within sample sites are plotted as pie plots on a map of the study area to show the geographic pattern of population structure (top), and individual assignment probabilities organized by basin are shown as a bar plot (bottom). A distinct subpopulation is found primarily in the Schuylkill River (orange), and four subpopulations are widely distributed throughout the Susquehanna River basin.

**Figure S4.** Results of the DAPC (discriminant analysis of principal component) clustering analysis for *K* = 9 subpopulations. Aggregated assignment probabilities within sample sites are plotted as pie plots on a map of the study area to show the geographic pattern of population structure (top), and individual assignment probabilities organized by basin are shown as a bar plot (bottom). A distinct subpopulation is found primarily in the Schuylkill River (orange), and eight subpopulations are widely distributed throughout the Susquehanna River basin, with some clusters (1, 2, 4, and 5) slightly more common in the Upper Susquehanna region and others (6, 7, and 8) slightly more common in the Lower Susquehanna near the river mouth.

**Table S1.** Selection of optimal number of *K* clusters in the Structure analysis using the four metrics from Puechmaille (2016) using Hydrological Unit Code 8 level river basins as a priori groups. *K* is the number of clusters assumed by Structure, and the values in each of the columns are the number of a priori groups, with mean (MedMeaK and MaxMeaK) or median (MedMedK and MaxMedK) assignment probabilities >0.5 for a unique cluster. Assignment probabilities were calculated across 10 replicate Structure runs, and the mean and median summary statistics are presented as the median (MedMeaK and MedMedK) or maximum (MaxMeaK and MaxMedK) number of a priori groups with unique cluster assignments across the 10 Structure‐run replicates.
**Table S2.** Selection of optimal number of *K* clusters in the Structure analysis using the four metrics from Puechmaille (2016) using sample sites as a priori groups. *K* is the number of clusters assumed by Structure, and the values in each of the columns are the number of a priori groups, with mean (MedMeaK and MaxMeaK) or median (MedMedK and MaxMedK) assignment probabilities >0.5 for a unique cluster. Assignment probabilities were calculated across 10 replicate Structure runs, and the mean and median summary statistics are presented as the median (MedMeaK and MedMedK) or maximum (MaxMeaK and MaxMedK) number of a priori groups with unique cluster assignments across the 10 Structure run replicates.
**Table S3.** Raw output from the MIGRAINE model estimating parameters of a population expansion after a bottleneck. *N* is the number of gene copies so the number of diploid individuals is equal to *N*/2.
**Table S4.** Confusion matrix for the diyABC analysis, showing the proportion of the scenario simulations, was correctly (along diagonal) or incorrectly (off diagonal) assigned to each scenario by random forest.
