# Supplementary figures and images for: Population genetic structure and demographic history reconstruction of introduced flathead catfish (Pylodictis olivaris) in two US Mid‐Atlantic rivers
Source: J Fish Biol. 2024 Aug 12;105(6):1614–27. doi: 10.1111/jfb.15888 (PMC11650961; doi:10.1111/jfb.15888)

**a** Evanno's delta K

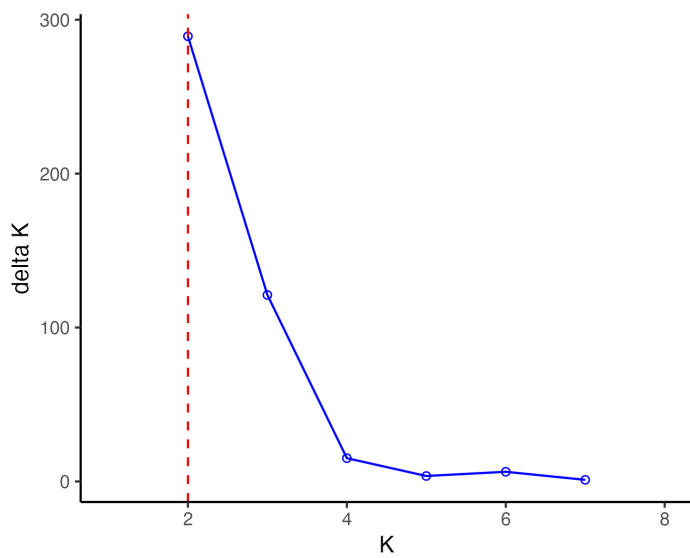

**b** Mean Log Likelihood

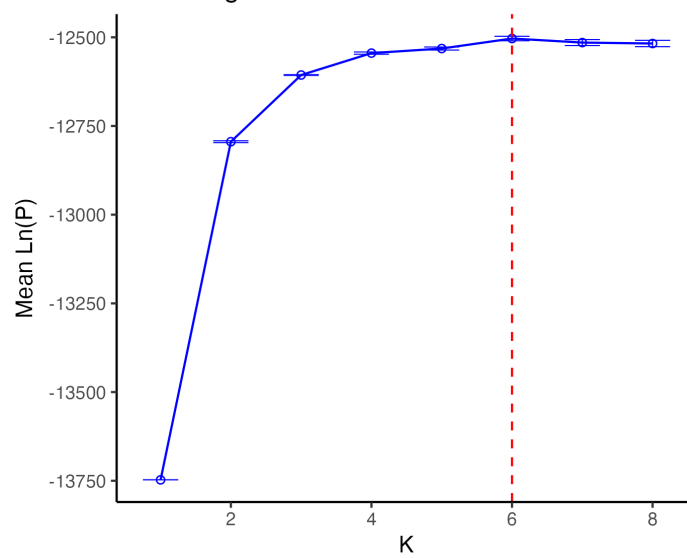

**c** DAPC K-Means BIC

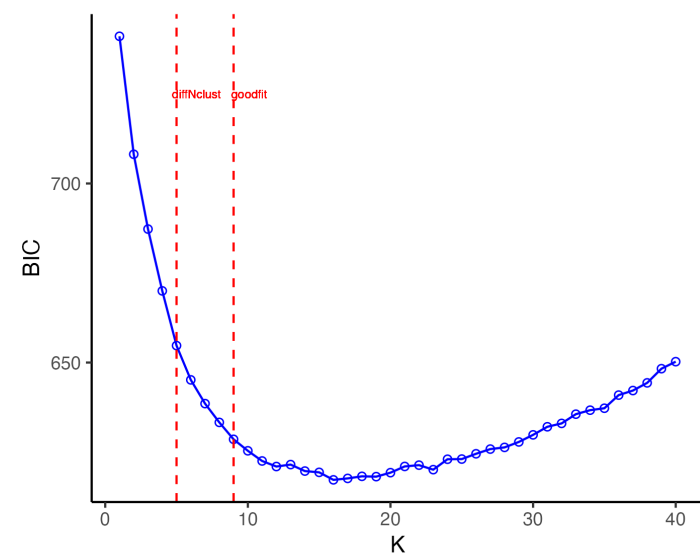

Supplement: Supplementary file 1 — Figure S1. Heuristics used to select the optimal number of K clusters from the Structure and DAPC (discriminant analysis of principal component) analyses, including the mean log likelihood along with SD across 10 independent runs of (a) Structure, (b) Evanno's ∆K measure, and (c) the Bayesian information criterion for k‐means clustering. The optimal number(s) of K are represented by vertical red dotted lines for each heuristic. [file JFB-105-1614-s003.pdf]

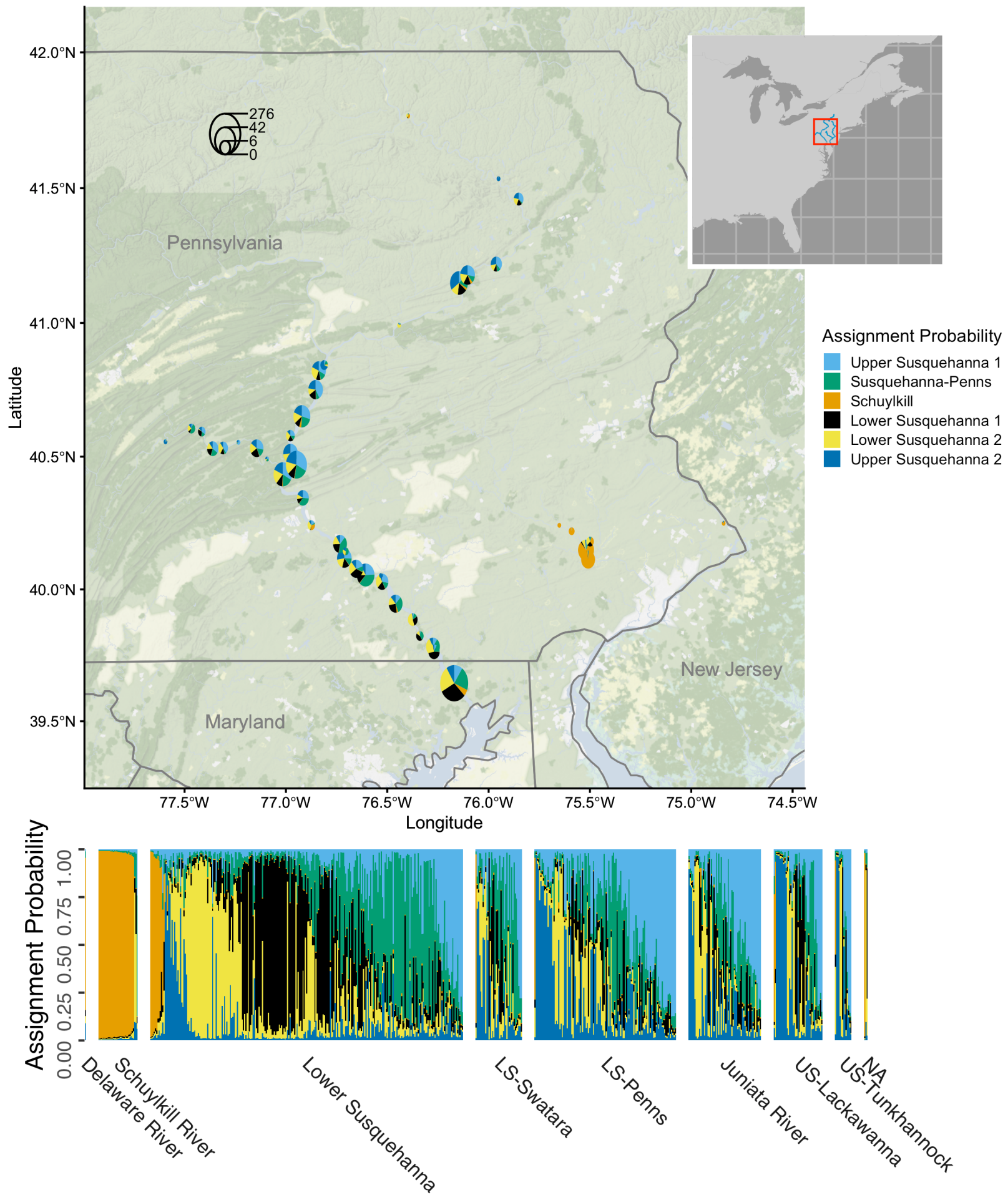

Supplement: Supplementary file 2 — Figure S2. Results of the Structure clustering analysis for K = 6 subpopulations. Aggregated assignment probabilities within sample sites are plotted as pie plots on a map of the study area to show the geographic pattern of population structure (top), and individual assignment probabilities organized by basin are shown as a bar plot (bottom). A distinct subpopulation is found primarily in the Schuylkill River (orange), and five subpopulations are widely distributed throughout the Susquehanna River basin. [file JFB-105-1614-s002.pdf]

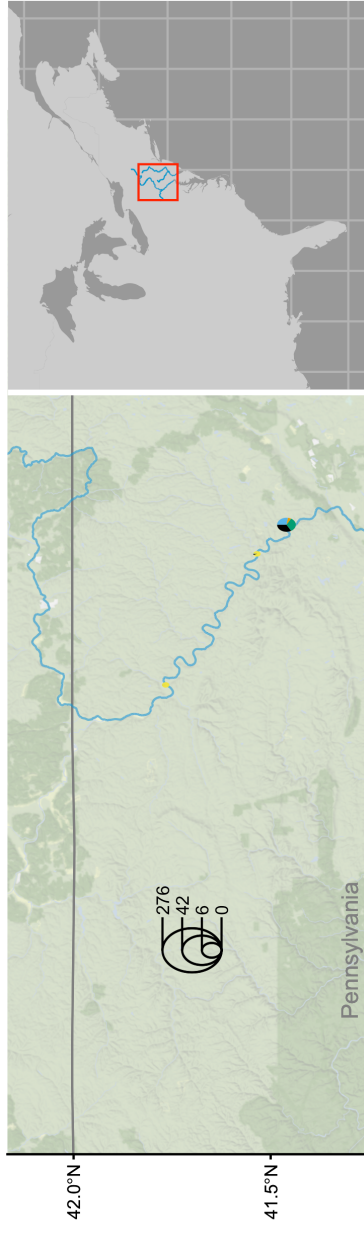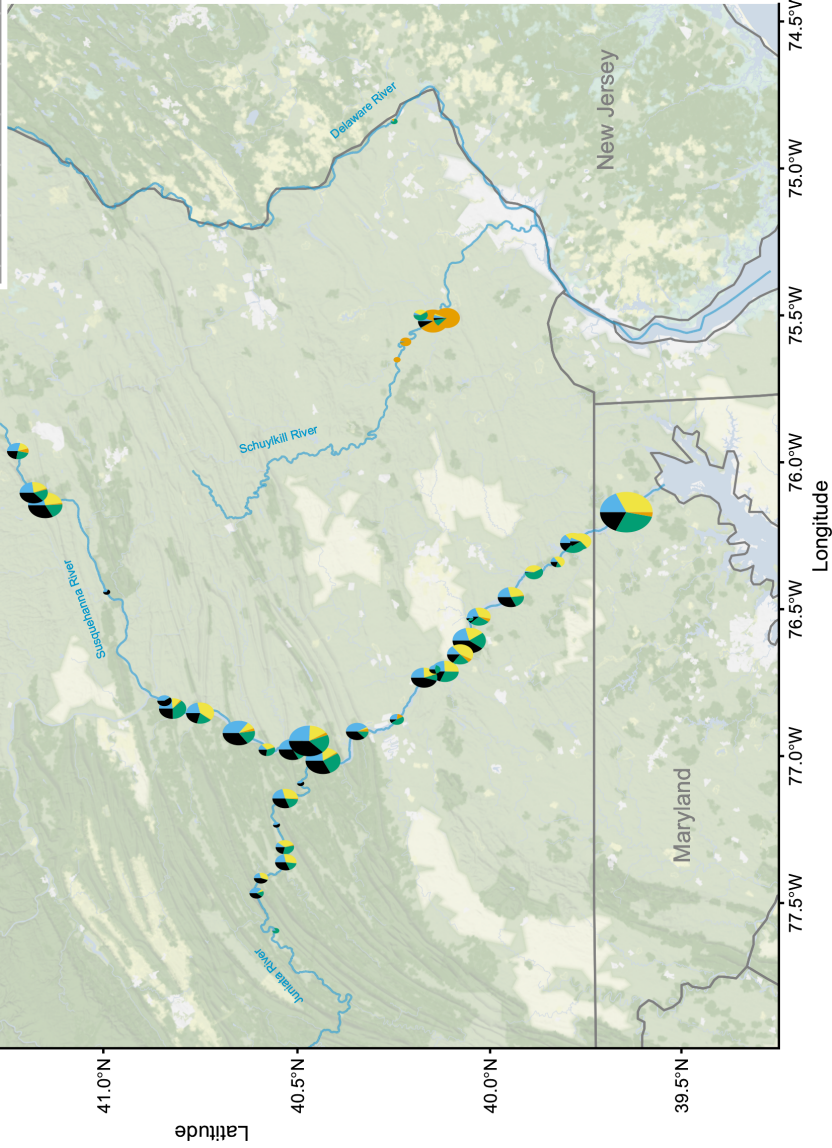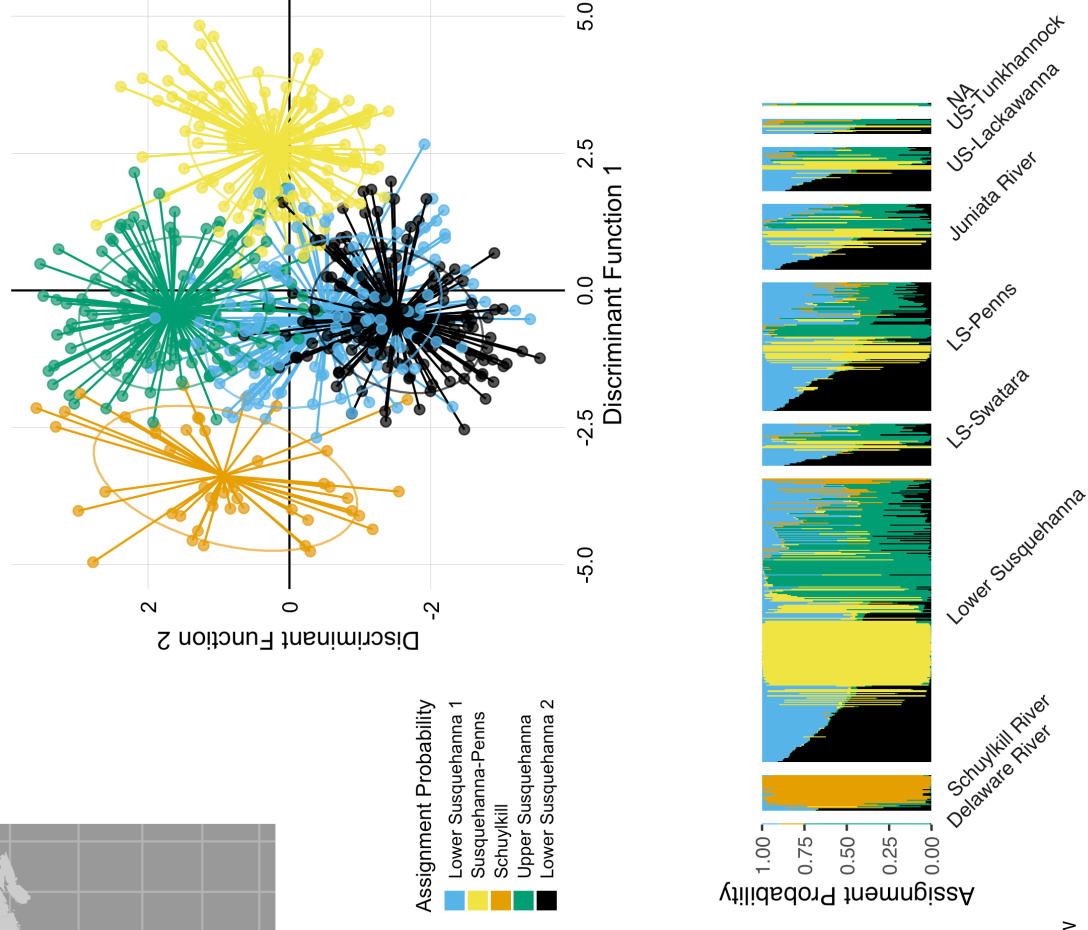

Supplement: Supplementary file 3 — Figure S3. Results of the DAPC (discriminant analysis of principal component) clustering analysis for K = 5 subpopulations. Aggregated assignment probabilities within sample sites are plotted as pie plots on a map of the study area to show the geographic pattern of population structure (top), and individual assignment probabilities organized by basin are shown as a bar plot (bottom). A distinct subpopulation is found primarily in the Schuylkill River (orange), and four subpopulations are widely distributed throughout the Susquehanna River basin. [file JFB-105-1614-s001.pdf]

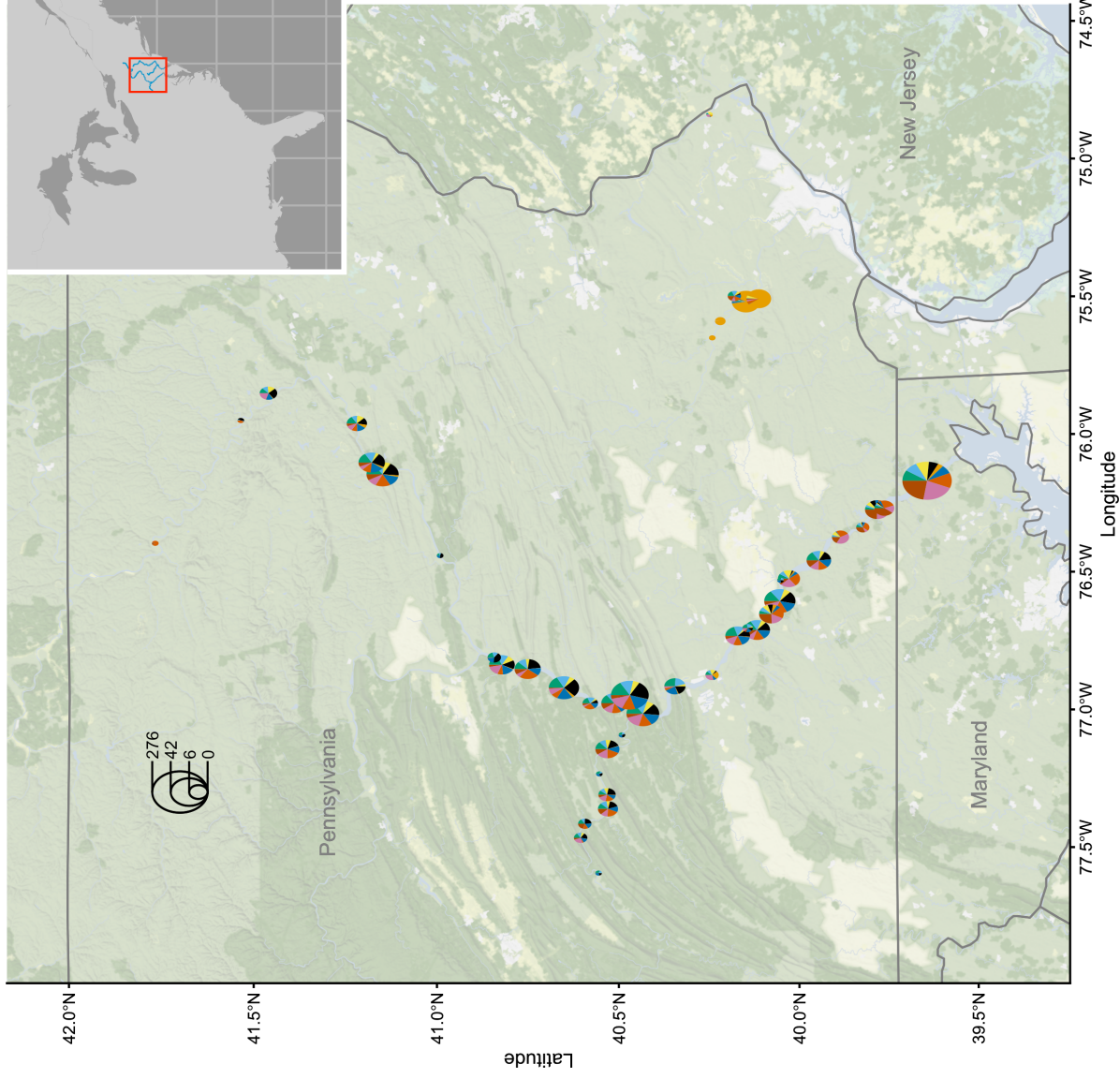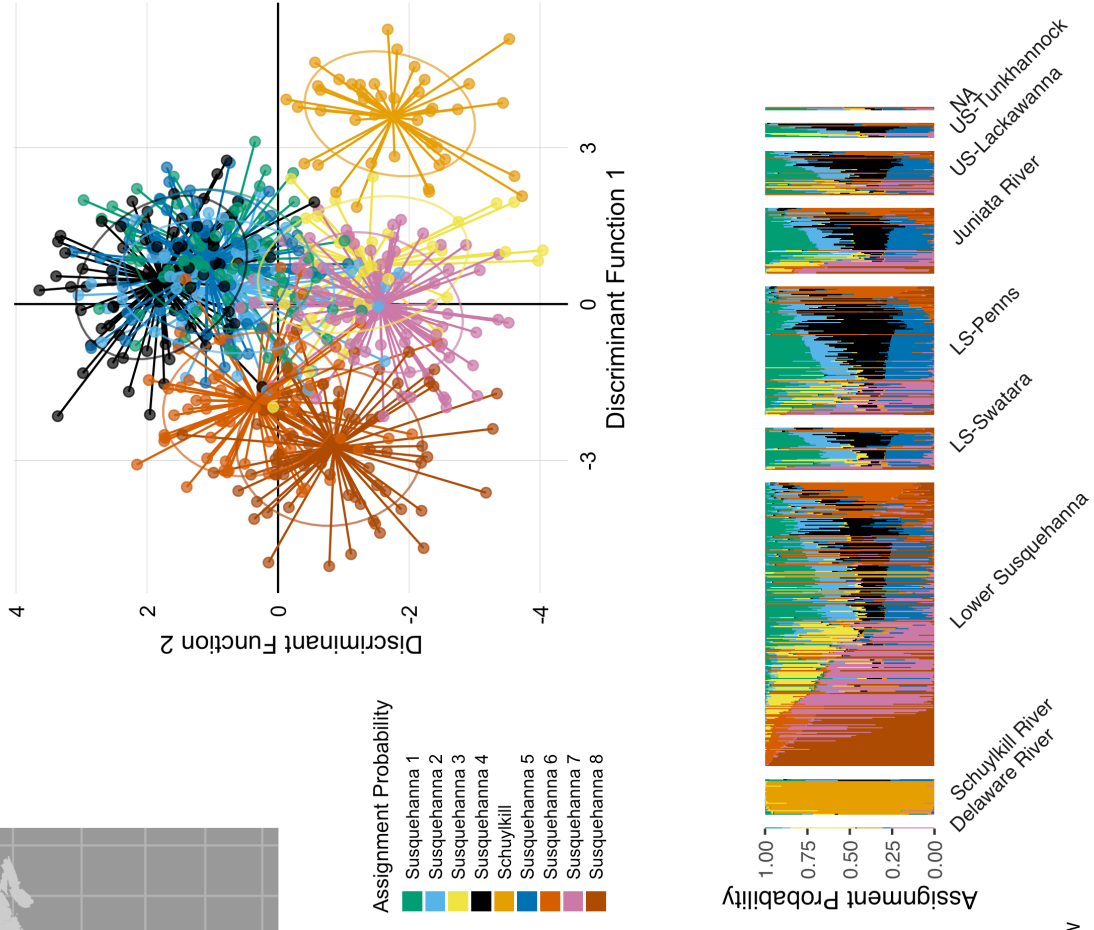

Supplement: Supplementary file 4 — Figure S4. Results of the DAPC (discriminant analysis of principal component) clustering analysis for K = 9 subpopulations. Aggregated assignment probabilities within sample sites are plotted as pie plots on a map of the study area to show the geographic pattern of population structure (top), and individual assignment probabilities organized by basin are shown as a bar plot (bottom). A distinct subpopulation is found primarily in the Schuylkill River (orange), and eight subpopulations are widely distributed throughout the Susquehanna River basin, with some clusters (1, 2, 4, and 5) slightly more common in the Upper Susquehanna region and others (6, 7, and 8) slightly more common in the Lower Susquehanna near the river mouth. [file JFB-105-1614-s005.pdf]
