# Supplementary material for: Population genetic structure and demographic history reconstruction of introduced flathead catfish (Pylodictis olivaris) in two US Mid‐Atlantic rivers
Source: J Fish Biol. 2024 Aug 12;105(6):1614–27. doi: 10.1111/jfb.15888 (PMC11650961; doi:10.1111/jfb.15888)
Supplement: Supplementary file 5 — Table S1. Selection of optimal number of K clusters in the Structure analysis using the four metrics from Puechmaille (2016) using Hydrological Unit Code 8 level river basins as a priori groups. K is the number of clusters assumed by Structure, and the values in each of the columns are the number of a priori groups, with mean (MedMeaK and MaxMeaK) or median (MedMedK and MaxMedK) assignment probabilities >0.5 for a unique cluster. Assignment probabilities were calculated across 10 replicate Structure runs, and the mean and median summary statistics are presented as the median (MedMeaK and MedMedK) or maximum (MaxMeaK and MaxMedK) number of a priori groups with unique cluster assignments across the 10 Structure‐run replicates. Table S2. Selection of optimal number of K clusters in the Structure analysis using the four metrics from Puechmaille (2016) using sample sites as a priori groups. K is the number of clusters assumed by Structure, and the values in each of the columns are the number of a priori groups, with mean (MedMeaK and MaxMeaK) or median (MedMedK and MaxMedK) assignment probabilities >0.5 for a unique cluster. Assignment probabilities were calculated across 10 replicate Structure runs, and the mean and median summary statistics are presented as the median (MedMeaK and MedMedK) or maximum (MaxMeaK and MaxMedK) number of a priori groups with unique cluster assignments across the 10 Structure run replicates. Table S3. Raw output from the MIGRAINE model estimating parameters of a population expansion after a bottleneck. N is the number of gene copies so the number of diploid individuals is equal to N/2. Table S4. Confusion matrix for the diyABC analysis, showing the proportion of the scenario simulations, was correctly (along diagonal) or incorrectly (off diagonal) assigned to each scenario by random forest. [file JFB-105-1614-s004.docx]

*Supplementary Tables*

Table S1. Selection of optimal number of K-clusters in the STRUCTURE analysis using the four metrics from Puechmaille (2016) using hydrological unit code level 8 river basins as a priori groups. K is the number of clusters assumed by STRUCTURE and the values in each of the columns are the number of a priori groups with mean (MedMeaK & MaxMeaK) or median (MedMedK & MaxMedK) assignment probabilities >0.5 for a unique cluster. Assignment probabilities were calculated across ten replicate STRUCTURE runs and the mean and median summary statistics are presented as the median (MedMeaK & MedMedK) or maximum (MaxMeaK & MaxMedK) number of a priori groups with unique cluster assignments across the ten STRUCTURE run replicates.

| K | MedMeaK | MaxMeaK | MedMedK | MaxMedK |
| --- | --- | --- | --- | --- |
| 1 | 1 | 1 | 1 | 1 |
| 2 | 2 | 2 | 2 | 2 |
| 3 | 3 | 3 | 3 | 3 |
| 4 | 2 | 2 | 2 | 2 |
| 5 | 1 | 1 | 1 | 1 |
| 6 | 1 | 1 | 1 | 1 |
| 7 | 1 | 1 | 1 | 1 |
| 8 | 1 | 1 | 1 | 1 |

Table S2. Selection of optimal number of K-clusters in the STRUCTURE analysis using the four metrics from Puechmaille (2016) using sample sites as a priori groups. K is the number of clusters assumed by STRUCTURE and the values in each of the columns are the number of a priori groups with mean (MedMeaK & MaxMeaK) or median (MedMedK & MaxMedK) assignment probabilities >0.5 for a unique cluster. Assignment probabilities were calculated across ten replicate STRUCTURE runs and the mean and median summary statistics are presented as the median (MedMeaK & MedMedK) or maximum (MaxMeaK & MaxMedK) number of a priori groups with unique cluster assignments across the ten STRUCTURE run replicates.

| K | MedMeaK | MaxMeaK | MedMedK | MaxMedK |
| --- | --- | --- | --- | --- |
| 1 | 1 | 1 | 1 | 1 |
| 2 | 2 | 2 | 2 | 2 |
| 3 | 3 | 3 | 3 | 3 |
| 4 | 4 | 4 | 4 | 4 |
| 5 | 4 | 5 | 4 | 5 |
| 6 | 6 | 6 | 6 | 6 |
| 7 | 3 | 4 | 3 | 4 |
| 8 | 2 | 4 | 2 | 4 |

Table S3. Raw output from the MIGRAINE model estimating parameters of a population expansion following a bottleneck. To convert the raw output to N is the number of gene copies so the number of diploid individuals is equal to N/2.

| Population | 2Nµ | Dg/2N | 2N_founder_µ | 2N_ancestral_µ | pGSM |
| --- | --- | --- | --- | --- | --- |
| Schuylkill River | 0.149  [0.0536-0.641] | 0.00000433  [0.00000113- 0.0000093] | 0.000253  [0.000166- 0.000502] | 1.143  [1.064- 1.19] | 0.619  [0.615-0.702] |
| Lower Susquehanna River | 1.036  [0.428-2.938] | 0.0316  [0.00594-0.214] | 0.0389 [0.0102-0.293] | 2.234  [0.788-7.979] | 0.537  [0.383-0.612] |
| Middle/Upper Susquehanna & Juniata Rivers | 0.846  [0.211-7.115] | 0.0131  [0.00071-0.199] | 0.00736  [0.00149-0.102] | 4.199  [1.012-16.91] | 0.396  [0.195-0.602] |

Table S3. Confusion matrix for the diyABC analysis, showing the proportion of the scenario simulations were correctly (along diagonal) or incorrectly (off diagonal) assigned to each scenario by the random forest.

|  | | Scenario assigned by random forest | | | | | | | |
| --- | --- | --- | --- | --- | --- | --- | --- | --- | --- |
|  |  | 1 | 2 | 3 | 4 | 5 | 6 | 7 | 8 |
| Simulated scenario | 1 | 0.472 | 0 | 0.006 | 0.250 | 0.251 | 0.010 | 0.009 | 0.001 |
|  | 2 | 0.001 | 0.945 | 0.011 | 0.001 | 0 | 0.001 | 0.004 | 0.036 |
|  | 3 | 0.018 | 0.016 | 0.538 | 0.008 | 0.007 | 0.131 | 0.138 | 0.145 |
|  | 4 | 0.186 | 0 | 0.001 | 0.434 | 0.350 | 0.010 | 0.019 | 0 |
|  | 5 | 0.170 | 0 | 0 | 0.362 | 0.443 | 0.013 | 0.012 | 0 |
|  | 6 | 0.015 | 0 | 0.074 | 0.015 | 0.016 | 0.458 | 0.359 | 0.061 |
|  | 7 | 0.025 | 0 | 0.052 | 0.040 | 0.039 | 0.342 | 0.453 | 0.050 |
|  | 8 | 0 | 0.036 | 0.117 | 0 | 0 | 0.065 | 0.086 | 0.696 |

*Supplemental Figures*

Figure S1. Heuristics used to select the optimal number of K clusters from the STRUCTURE and DAPC analyses including the mean log likelihood along with standard deviation across 10 independent runs of STRUCTURE (A), Evanno’s ∆K measure (B), and the Bayesian Information Criterion for k-means clustering (C). The optimal number(s) of K are represented by vertical red dotted lines for each heuristic.

Figure S2. Results of the STRUCTURE clustering analysis for K=6 subpopulations. Aggregated assignment probabilities within sample sites are plotted as pie plots on a map of the study area to show the geographic pattern of population structure (top) and individual assignment probabilities organized by basin are shown as a bar plot (bottom). There is a distinct subpopulation found primarily in the Schuylkill River (orange) and five subpopulations widely distributed throughout the Susquehanna River basin.

Figure S3. Results of the DAPC clustering analysis for K=5 subpopulations. Aggregated assignment probabilities within sample sites are plotted as pie plots on a map of the study area to show the geographic pattern of population structure (top) and individual assignment probabilities organized by basin are shown as a bar plot (bottom). There is a distinct subpopulation found primarily in the Schuylkill River (orange) and four subpopulations widely distributed throughout the Susquehanna River basin.

Figure S4. Results of the DAPC clustering analysis for K=9 subpopulations. Aggregated assignment probabilities within sample sites are plotted as pie plots on a map of the study area to show the geographic pattern of population structure (top) and individual assignment probabilities organized by basin are shown as a bar plot (bottom). There is a distinct subpopulation found primarily in the Schuylkill River (orange) and eight subpopulations widely distributed throughout the Susquehanna River basin, with some clusters (1, 2, 4, and 5) slightly more common in the upper Susquehanna region and others (6, 7, and 8) slightly more common in the lower Susquehanna near the river mouth.
